# Effect of hypothermia on coagulation functions in Sprague-Dawley rats with uncontrolled hemorrhagic shock

**DOI:** 10.1186/cc9859

**Published:** 2011-03-11

**Authors:** KH Park

**Affiliations:** 1 Jeju Natioanl University Hospital, Jeju, South Korea

## Introduction

Acute coagulopathy, hypothermia, and acidosis are known as the lethal triad of major trauma patients. Major trauma patients with hypothermia and acidosis developed clinically significant bleeding despite adequate transfusion [1]. Recent animal experiment studies reported that hypothermia is associated with improved survival in controlled hemorrhagic shock [2,3]. Post-traumatic hypothermia is an unproven therapy unlike hypothermia as a postcardiac arrest care. The objective of this study was to investigate the effect of hypothermia on coagulation function in uncontrolled hemorrhagic shock with major trauma.

## Methods

Thirty-two male Sprague-Dawley rats were divided into four groups randomly: Group 1 with normothermia (control, 37 to 38°C); Group 2 with hypothermia (33 to 34°C on rectal temperature); Group 3 with hypothermic hemorrhagic shock; Group 4 with normothermic hemorrhagic shock. Hemorrhagic shock was induced by splenic laceration or blood shedding. Coagulation functions were measured by rotation thrombelastometry (ROTEM^®^). The clotting time, clot formation time (CFT), and maximum clot firmness (MCF) were measured at baseline, after 1 hour shock, and after 1 hour resuscitation. They were compared among the four groups using the Kruskal-Wallis test with Bonferroni correction, and the Friedman test was used to detect the differences in the repeated measures in the same group, taking *P *< 0.05 as a significant level.

## Results

No significant differences showed among the groups at baseline. CFT after the shock period of group 2 was longer than that of group 4. MCF after resuscitation of group 2 was higher than that of groups 3 and 4. When the factors were compared as a time process, CFT and MCF after shock and resuscitation of group 3 decreased significantly compared with baseline. Four in group 3 and two in group 4 died during 48 hours of observation.

## Conclusions

Although it took a shorter time to form a clot in normothermic shock compared with hypothermia, clot firmness was poorer in hemorrhagic shock. In addition, clot firmness was significantly worse in the shock period and after resuscitation in the hypothermic shock group. Only hypothermia does not deteriorate coagulation, but hypothermia combined with hemorrhagic shock deteriorates coagulation.
